# Effective Recruitment Strategies for a Sickle Cell Patient Registry Across Sites from the Sickle Cell Disease Implementation Consortium (SCDIC)

**DOI:** 10.1007/s10903-020-01102-6

**Published:** 2020-10-09

**Authors:** Rita V. Masese, Terri DeMartino, Emily Bonnabeau, Ebony N. Burns, Liliana Preiss, Taniya Varughese, Judith M. Nocek, Patricia Lasley, Yumei Chen, Caroline Davila, Chinonyelum Nwosu, Samantha Scott, Latanya Bowman, Lauren Gordon, Cindy Clesca, Marlene Peters-Lawrence, Cathy Melvin, Nirmish Shah, Paula Tanabe, SCD Implementation Consortium

**Affiliations:** 1 Duke University School of Nursing, DUMC 3322, 307 Trent Drive, Durham, NC 27710 USA; 2 RTI International, Durham, NC USA; 3 Program in Occupational Therapy, Washington University School of Medicine, St. Louis, MO USA; 4 Department of Medicine, Comprehensive Sickle Cell Center, University of Illinois at Chicago, Chicago, IL USA; 5 Sinai Health System, Chicago, IL USA; 6 University of California, San Francisco, Benioff Children’s Hospital Oakland, 747 52nd Street, Oakland, CA 94609 USA; 7 Medical University of South Carolina, Charleston, SC USA; 8 St. Jude Children’s Research Hospital, Memphis, TN USA; 9 Augusta University Center for Blood Disorders, Augusta, GA USA; 10 Department of Emergency Medicine, Icahn School of Medicine at Mount Sinai, New York, USA; 11 Division of Blood Diseases and Resources, National Institute of Health, National Heart Lung and Blood Institute, Bethesda, MD USA

**Keywords:** Recruitment, Sickle cell disease, Multi-site studies, Minority populations, Barriers

## Abstract

Sickle cell disease (SCD) is a genetic disorder predominantly affecting people of African descent and is associated with significant morbidity and mortality. To improve SCD outcomes, the National Heart Lung and Blood Institute funded eight centers to participate in the SCD Implementation Consortium. Sites were required to each recruit 300 individuals with SCD, over 20 months. We aim to describe recruitment strategies and challenges encountered. Participants aged 15–45 years with confirmed diagnosis of SCD were eligible. Descriptive statistics were used to analyze the effectiveness of each recruitment strategy. A total of 2432 participants were recruited. Majority (95.3%) were African American. Successful strategies were recruitment from clinics (68.1%) and affiliated sites (15.6%). Recruitment at community events, emergency departments and pain centers had the lowest yield. Challenges included saturation of strategies and time constraints. Effective recruitment of participants in multi-site studies requires multiple strategies to achieve adequate sample sizes.

## Introduction

Participant recruitment is an integral component of research studies involving human subjects. Adequate recruitment has proven to be challenging, particularly in studies focusing on conditions that predominantly affect minority populations such as sickle cell disease (SCD) [1–4]. SCD is a complex, chronic, genetic blood disorder that primarily affects people of African descent [5, 6]. In the United States, 1 in 396 African Americans has SCD, and one in 14 carry the trait [5]. The disease has several complications including severe pain episodes, acute chest syndrome and stroke which lead to frequent hospitalization, poor quality of life and early mortality [7–9]. The median lifespan for individuals with SCD is 42 years for males and 48 years for females [9]. In addition to the medical complexities of the disease, people with SCD encounter significant barriers to healthcare access and provision such as lack of insurance, lack of transportation to health facilities, stigmatization by healthcare providers, limited provider knowledge, and poor care coordination and transition within the healthcare system [6, 10]. Due to the complexity of the disease, it is important to conduct research aimed at developing novel SCD therapies or interventions tailored towards addressing the numerous social, economic, and healthcare barriers faced by individuals with SCD [11]. However, for these research interventions to be effective, adequate participant recruitment is required [4, 12].

There are several barriers to SCD participant recruitment, which include distrust of research, challenging life situations, debilitating chronic pain, stigma and logistic challenges such as child or elder care and transportation [13, 14]. Due to these barriers, research teams are often challenged to achieve intended sample sizes and have to extend recruitment periods or adjust study inclusion criteria [15, 16]. This often results in increased study workload and costs, delays in initiating potentially effective interventions or premature termination of research studies, all of which negatively affect medical advancement in SCD and contribute to the ongoing disparities in healthcare provision and research funding [15, 16].

Published literature on recruitment strategies utilized in SCD studies has identified that a patient-centered approach and clinical staff buy-in positively impact recruitment [17–19]. Majority of those studies, however, focused on the pediatric SCD population [17–19]. To address this gap, we examined recruitment strategies across sites participating in the Sickle Cell Disease Implementation Consortium (SCDIC) registry project [20]. In addition to describing the recruitment strategies utilized by the SCDIC, we will also outline challenges encountered during the recruitment process and subsequent strategy adaptations. These findings may help inform recruitment strategies in future multi-site SCD research studies and other studies involving minority populations.

## Methods

### Study Setting

The SCDIC is composed of eight academic SCD centers across the US and one data coordinating center [20]. The sites are representative of the geographic and urban diversity in the distribution of sickle cell patients in the US (Table 1). Seven of the eight sites offer adult and pediatric care, the other offers pediatric care (Table 1). The consortium was established in 2016, by the National Heart Lung and Blood Institute with the goal of improving the quality of SCD care delivery and to develop a longitudinal registry of individuals with SCD [20]. The registry was created as a resource for conducting data queries and identifying gaps in research that may inform interventions tailored towards addressing barriers to SCD care.

**Table 1:** Description of study sites

Site	Geographic region in the US	Estimated number of individuals with SCD reviewed at the SCDIC site	Participant Compensation for enrollment in the SCDIC registry in USD	Time taken to achieve recruitment target (months)
Site 1	Southeast	~ 1568 adults and adolescents	20	13
Site 2	Midwest	~ 600 adults and adolescents	25	16
Site 3	Southeast	~ 500 adults and 150 adolescents	20	16
Site 4	Northeast	~ 284 adults and adolescents	25	18
Site 5	Southeast	~ 450 adults and 120 adolescents	20	14
Site 6^a^	Southeast	~ 590 adolescents and young adults	0	15
Site 7	West	~ 500 adolescents and adults	100	15
Site 8	Midwest	~ 350 adults and 60 adolescents	30	19

^a^Site 6 primarily offers pediatric care

Recruitment for the SCDIC patient registry occurred in the eight comprehensive sickle cell disease centers. The minimum recruitment number was set at 300 per site in order to achieve a target of approximately 2400 participants. Sites were expected to recruit participants over twenty months.

### Ethics Statement

Ethical approval was received from the Institutional Review Boards (IRBs) at the eight participating sites prior to any recruitment efforts. Participant compensation was guided by the SCDIC protocol and ranged from 0 to 100 USD (Table 1). The exact amount was left at the discretion of the sites, as their IRBs permitted. Majority of the sites offered $20-$30, site 7 offered $100 commensurate to the high cost of living in that area.

### Eligibility Criteria

Participants were eligible for inclusion in the SCDIC registry if they met the following criteria: (1) had a confirmed diagnosis of SCD (subtypes Hb SS, SC, Sβ-thalassemia, SO, SD or SE; (2) were between the ages of 15–45; (3) were literate in English; and (4) were willing to provide informed consent or assent and complete a patient enrollment survey. SCD diagnosis was confirmed by written documentation of a positive SCD test result from the participants’ medical records or if the enrolling center conducted their own confirmatory laboratory test. Participants were excluded from enrollment if they were unwilling or unable to provide informed consent or assent, unable to complete the patient enrollment survey, had sickle cell trait (Hb AS) or had a successful bone marrow transplant.

### Recruitment Process and Strategies Utilized

Research coordinators at each site were the key personnel responsible for recruiting participants. In order to raise awareness about the study, flyers were placed in the adult and pediatric sickle cell clinics, advertisements were posted on social media sites and letters were sent to eligible participants who had consented to be contacted about research studies conducted at their medical institution. Recruitment strategies included making targeted phone calls to eligible participants and approaching and consenting eligible participants during outpatient clinic appointments, inpatient hospital admissions, at community events, or other platforms approved by the local IRB. Research coordinators also used word of mouth and snowballing to enroll additional participants. Some sites contacted churches and historically black colleges and universities in their area to promote awareness of the study.

### Data Collection

After obtaining informed consent, participants completed an enrollment survey electronically or on paper. The enrollment survey was developed by the SCDIC steering committee; consisting of at least one member from each of the sites. Coordinators at each study site documented and tracked the number of participants enrolled through each recruitment strategy into a centralized recruitment tracking system developed by the data-coordinating center. The data-coordinating center also facilitated monthly multi-center coordinator meetings to discuss recruitment challenges and successes. Extensive notes were taken during the meetings, and strategy adaptations were discussed.

### Data Analysis

Patient demographic data from the enrollment survey were entered into a Research Electronic Data Capture (REDCap) database [21]. SAS statistical software (version 9.4) was used to conduct descriptive analyses that determined percentages, means and standard deviations where appropriate. Documentation of the recruitment procedures and meeting notes from monthly coordinator meetings were analyzed to determine the types of recruitment strategies and the success rates.

## Results

From July 2017 to February 2019, 2432 individuals with SCD were recruited into the SCDIC registry. The mean age of our participants was 28.1 years (SD ± 7.9). Almost all (95.3%) were black or African American. Slightly more than half (56.9%) were female and had sickle cell phenotype Hb SS (69.3%). A summary of participant demographics by recruitment site is highlighted in Table 2. Recruitment strategies utilized to obtain our sample size are outlined in Table 3.

**Table 2:** Participant demographics

	All sites	Site 1	Site 2	Site 3	Site 4	Site 5	Site 6	Site 7	Site 8
	N = 2432	N = 300	N = 304	N = 305	N = 300	N = 306	n = 302	N = 309	N = 306
	N (%)/mean (SD)	N (%)/mean (SD)	N (%)/mean (SD)	N (%)/mean (SD)	N (%)/mean (SD)	N (%)/mean (SD)	N (%)/mean (SD)	N (%)/mean (SD)	N (%)/mean (SD)
Age									
Age in years, mean (SD)	28.1 (7.9)	29 (8.1)	29.8 (7.1)	27.7 (7.8)	30.8 (6.6)	28.3 (7.3)	22.9 (7.4)	28.6 (8.6)	27.4 (7.6)
Age categories n (%)									
15–24	894 (36.8)	94 (31.3)	75 (24.7)	117 (38.4)	55 (18.3)	106 (34.6)	211 (69.9)	114 (36.9)	122 (39.9
25–34	976 (40.1)	121 (40.3)	144 (47.4)	128 (42.0)	152 (50.7)	138 (45.1)	63 (20.9)	105 (34.0)	125 (40.8)
35–45	562 (23.1)	85 (28.3)	85 (28.0)	60 (19.7)	93 (31.0)	62 (20.3)	28 (9.3)	90 (29.1)	59 (19.3)
Sex n (%)									
Female	1369 (56.9)	170 (56.7)	176 (57.9)	173 (58.2)	160 (53.9)	193 (63.3)	141 (48.8)	177 (57.7)	179 (58.5)
Male	1036 (43.1)	130 (43.3)	128 (42.1)	124 (41.8)	137 (46.1)	112 (36.7)	148 (51.2)	130 (42.3)	127 (41.5)
Race n (%)									
Black or African American	2237 (95.3)	288 (97.0)	290 (96.3)	282 (96.2)	249 (94.7)	294 (96.7)	276 (96.5)	270 (90.0)	288 (95.0)
Multi-racial	80 (3.4)	7 (2.4)	8 (2.7)	8 (2.7)	5 (1.9)	10 (3.3)	6 (2.1)	24 (8.0)	12 (4.0)
Other	30 (1.3)	2 (0.7)	3 (1.0)	3 (1.0)	9 (3.4)	0	4 (1.4)	6 (2.0)	3 (1.0)
Sickle cell genotype n (%)									
Hb SS	1675 (69.3)	220 (73.3)	216 (71.8)	203 (67.9)	200 (67.8)	225 (73.5)	195 (64.6)	226 (73.1)	190 (62.1)
Hb SC	498 (20.6)	54 (18.0)	54 (17.9)	66 (22.1)	63 (21.4)	56 (18.3)	70 (23.2)	56 (18.1)	79 (25.8)
Hb S beta + thalassemia	131 (5.4)	15 (5.0)	18 (6.0)	15 (5.0)	14 (4.7)	18 (5.9)	17 (5.6)	18 (5.8)	16 (5.2)
Hb S beta0 thalassemia	89 (3.7)	10 (3.3)	11 (3.7)	12 (4.0)	16 (5.4)	4 (1.3)	17 (5.6)	6 (1.9)	13 (4.2)
Hb S hereditary persistence of fetal Hb(S/HPFH)	14 (0.6)	0	1 (0.3)	1 (0.3)	2 (0.7)	1 (0.3)	2 (0.7)	0	7 (2.3)
Hb SO	4 (0.2)	1 (0.3)	0	1 (0.3)	0	0	0	2 (0.6)	0
Hb SD	6 (0.2)	0	1 (0.3)	1 (0.3)	0	2 (0.7)	1 (0.3)	0	1 (0.3)
Hb SE	1 (0.0)	0	0	0	0	0	0	1 (0.3)	0

**Table 3:** Recruitment strategies and yield across the eight Sickle Cell Disease Implementation Consortium sites

	All sites	Site 1	Site 2	Site 3	Site 4	Site 5	Site 6^c^	Site 7	Site 8^e^
	N (%)	N (%)	N (%)	N (%)	N (%)	N (%)	N (%)	N (%)	N (%)
Clinic visit	1650 (68.1)	239 (79.7)	201 (66.8)	209 (69.9)	148 (49.3)	305 (100.0)	109 (36.1)	170 (55.0)^d^	269 (87.9)
Affiliated sites	377 (15.6)	61 (20.3)	1 (0.3)	31 (10.4)	55 (18.3)	0	166 (55.0)	61 (19.7)	2 (0.7)
Targeted phone calls	144 (5.9)	0	0	47 (15.7)	35 (11.7)	0	2 (0.7)	56 (18.1)	4 (1.3)
Hospital in-patient	141 (5.8)	0	68 (22.6)	12 (4.0)	32 (10.7)	0	10 (3.3)	0	19 (6.2)
Community event	52 (2.1)	0	5 (1.7)	0	8 (2.7)	0	8 (2.6)	19 (6.1)	12 (3.9)
Pain/infusion center	25 (1.0)	0	22 (7.3)	0	1 (0.3)	0	0	2 (0.6)	0
Emergency department	14 (0.6)	0	1 (0.3)	0	13 (4.3)	0	0	0	0
Other^a^	19 (0.8)	0	3 (1.0)	0	8 (2.7)	0	7 (2.3)	1 (0.3)	0
Total (non-missing responses)	2,422	300	301	299	300	305	302	309	306
Missing^b^	10	0	3	6	0	1	0	0	0

^a^Other refers to recruitment at other medical visits and in-person interviews

^b^Missing refers to survey items with no response

^c^Site 6 approached 386 individuals overall, 302 individuals were enrolled

^d^Site 7 approached 173 individuals in the clinic, 3 declined due to various reasons: (1) not interested in the study, (2) time constraints, (3) a minor and their parents declined participation)

^e^Site 8 approached 312 people overall, 5 declined participation, one was not eligible, 306 individuals consented and were enrolled

### Primary Strategy: Recruitment in the Adult and Pediatric Clinics

The first strategy was in-person recruitment from the adult and pediatric SCD clinics, which accounted for 68.1% of the sample. Research coordinators reviewed the clinic schedule, identified eligible participants and created a patient list. The coordinators worked with the clinic staff to ensure that healthcare providers were aware of eligible registry participants. Healthcare providers would introduce the study to eligible participants and refer them to the research coordinators after their clinic appointment. Although this strategy proved to be very successful, coordinators reported the following challenges with clinic recruitment: (1) disruption of clinic work flow; (2) providers forgetting to introduce the study to eligible participants; (3) high rate of eligible participants not showing up for their clinic appointments; (4) individuals reporting lack of time to complete study procedures; (5) limited research personnel to complete study procedures with all eligible participants; (6) lack of space or rooms in the clinic for recruitment activities; and (7) competing SCD related research studies. For individuals who consented to the study but were unable to complete study procedures due to time constraints, research staff emailed links to the online survey, approached them in their next clinic visit or scheduled a phone meeting. Despite its high recruitment yield, in-clinic recruitment proved to be unsuccessful at capturing people who were lost to clinic follow-up, received most of their care in the emergency department, or were unaffiliated with the study site. Only one site (Site 5) was able to achieve nearly all (99.7%) recruitment from in-person clinic visits.

### Secondary Strategies

As recruitment at the main sites approached saturation (about 6–8 months after enrollment opened), where the research team had already approached almost all eligible participants, sites shifted their efforts to recruiting in alternative venues. Most sites began recruiting at their affiliated sites and inpatient care. Affiliated sites were defined as community hospitals in the same state as the primary SCDIC study sites that were willing to refer participants to the SCDIC site. In parallel, sites also began recruiting at pain/infusion centers, the emergency department and at community events.

### Recruitment from Affiliated Sites

Recruitment from affiliated sites was the second most successful strategy, accounting for 15.6% of the total sample, with varying degrees of success across the eight sites. While site 6 found this strategy to be highly successful in recruiting 55% of their participants, three sites (sites 2, 5, and 8) found it to be highly unsuccessful. With the exclusion of site 6, recruitment from affiliated sites remained the second most successful strategy accounting for 10% of the overall sample. The primary challenge associated with recruiting people outside of the primary site was the difficulty in obtaining SCD diagnosis confirmation. To resolve this challenge, the SCDIC research teams had to coordinate with the affiliated sites to obtain confirmation of the participants’ disease status. This often required back and forth communication, travel and additional paperwork.

### Inpatient Recruitment

Inpatient recruitment accounted for 5.8% of the sample and was utilized by five sites. (Site 2, 3, 4, 6 and 8). Research coordinators approached eligible participants during their hospitalizations when they were near their steady state of health. Caution was taken whenever a participant was asked to complete study procedures as an inpatient, since it was difficult to determine if participants were truly at their steady state prior to hospital discharge. Oftentimes coordinators would meet with the patient to introduce the study and follow-up with them later to complete study procedures. One benefit of this strategy was the absence of time restrictions for the consent and enrollment process. Participants did not feel rushed to complete the survey and coordinators could leave the survey with the participant to complete at their own pace.

### Recruitment from the Emergency Department and Pain/Infusion Center

Recruitment in the emergency department and the pain/infusion center accounted for a very small proportion of the sample (0.6% and 1.0% respectively) and was only utilized by three sites, after saturation of clinic and inpatient recruitment.

### Targeted Phone Calls and Opt-Out Letters

Targeted phone calls accounted for 5.9% of the sample and was used by five centers. This strategy was utilized after saturation of clinic, inpatient and affiliated site recruitment. The research coordinators compiled a list of participants who were lost to clinic follow-up. Two centers (site 3 and 7) sent out letters to eligible participants prior to making calls. The letter informed participants that a study coordinator would contact them for research participation. Potential participants could “opt out” of being contacted. Two weeks after receipt of the opt-out letters, research coordinators called people on the list. An IRB approved phone script was utilized to inform eligible participants about the study. Interested participants provided verbal consent and completed the enrollment survey verbally through the phone, via e-mail or scheduled a time to come to the clinic to complete the survey. Although coordinators continually updated phone numbers with the most up to date information from the patient’s medical chart, a small number of individuals with disconnected or unreliable phone service were unable to be reached.

### Recruitment at Community Events

Research teams at five sites (sites 2, 4, 6, 7, and 8) recruited eligible participants at community events (SCD conferences, support groups, health fairs and SCD walks). Coordinators set up tables at events to recruit and enroll eligible participants or distribute flyers. Challenges associated with this strategy were (1) the same group of individuals attended most of the community events so the pool of eligible participants dwindled quickly; and (2) inability to obtain SCD confirmation from participants not associated with the study site or their affiliated sites. This strategy accounted for only 2.1% of the sample.

## Discussion

The aim of this paper was to identify recruitment strategies utilized by sites participating in the SCDIC and describe the challenges encountered. Overall, the strategies used were highly successful: all sites were able to meet the recruitment goal of 300 participants within the required timeline. The strategy with the highest yield in almost all sites was recruitment in the adult and pediatric clinics. Successful recruitment from this strategy may be attributed to the well-established, academic nature of the sites, each with a large patient population accustomed to research. Similar to other studies, initial contact and introduction of the study was facilitated by healthcare providers and clinical staff with established trusting relationships with research participants [16, 22, 23]. Pre-study clinic visits were helpful to establish rapport and collaboration between the research team and clinic staff [24]. These visits allowed for the clinic staff to understand and discuss study recruitment procedures and for the research team to better understand clinic work flow, patient volumes and administrative protocols that govern the clinic [22]. For effective recruitment, clinic staff need to know exactly how much time recruitment will take and what information they need to provide to participants prior to any recruitment efforts [22].

Similar to other studies, our results indicate that several strategies need to be utilized to attain recruitment goals [16, 22, 23, 25]. Despite being the strategy with the highest yield in almost all sites, recruitment in the adult and pediatric clinics reached saturation, where the research team had already approached almost all eligible participants. Frequent communication between the SCDIC sites facilitated the adaptation of other recruitment strategies. Recruitment from inpatient service, affiliated clinics, targeted phone calls and opt-out letters accounted for approximately a quarter (25%) of the sample. The use of several strategies assisted in overcoming barriers to recruit African Americans and other minority populations as noted in previous literature [16, 24, 26, 27]. The shortcomings of one strategy were compensated for by another. Participants who could not be recruited in the clinic due to transportation barriers, time constraints or child and elder care could be recruited via targeted phone calls and opt-out letters.

Although we did not directly factor in the cost and labor associated with each strategy, coordinators from each site identified recruitment from affiliated sites and community events to be the most resource and labor-intensive due to the paperwork and communication required to confirm SCD diagnosis. An exception to this was site 6 which achieved more than half (55%) of target recruitment from affiliated sites. This can be attributed to the long-standing relationship between the primary and affiliated sites. Site 6 is a pediatric hospital and transitions youth to adult care in the affiliated sites. This also accounts for the high proportion of 15–24 year olds in that site when compared to the others. Targeted phone calls and direct mailing were the most cost-effective and least labor-intensive strategies. Factors such as cost, labor, expertise and experience need to be accounted for in determining the appropriate recruitment strategy [22, 28]. Our findings suggest that, for participants who visit clinics infrequently, recruitment via targeted phone calls and direct mailing are more successful than via community events. Recruitment at community events was among the least successful strategies accounting for only 2% of the sample.

In addition to the strategies outlined above, a unique feature of the SCDIC that may have contributed to recruitment success was having one data-coordinating center for all the sites. The center facilitated a monthly meeting to discuss recruitment successes and failures, which served as educational and motivational sessions for coordinators. The center also created a centralized, comprehensive electronic recruitment management system that enabled research teams to recruit, track and retain enrolled participants [21].

Our paper is not without limitations. Most sites were not able to adequately document the number of participants who were approached and declined participation and the reasons why they declined. However, coordinators noted that most subjects were willing to participate, with only a handful of participants from each site declining to participate. Prior to enrollment, one site (Site 2) interviewed 40 potential participants about their likelihood of joining the registry. Altruism and a desire to increase knowledge was noted as a motivator for participation by 31 of the 40 subjects. The most common reasons for declining to participate were inconvenient timing, concerns about privacy, and issues relating to the recruiter’s approach. Additional reasons for declining to participate that were noted in other sites were distrust or lack of interest in research, or concurrent participation in other studies.

Although we did not formally document the resources associated with each strategy, we identified recruitment at the clinic, targeted phone calls and opt-out letters as the most cost-effective strategies in terms of staff time per enrolled participant. Majority of our enrolled participants were associated with a health care system. Our approaches may not be generalizable to other studies recruiting participants from the general population. Despite these limitations, this study provides useful insights on recruitment strategies in SCD studies, multi-site studies, and studies involving minority populations.

## Conclusion

Effective recruitment of participants in multi-site SCD studies requires the adaptation of various strategies and a centralized recruitment management system in order to achieve target sample sizes. The use of multiple recruitment strategies ultimately led to successful recruitment, and was required to overcome challenges.
